# Impact of perioperative blood transfusions on postoperative renal function and survival after resection of colorectal liver metastases

**DOI:** 10.1186/s12957-022-02559-5

**Published:** 2022-03-30

**Authors:** Wiebke Rodieck, Michael Hallensleben, Julia Robert, Oliver Beetz, Gerrit Grannas, Sebastian Cammann, Felix Oldhafer, Juergen Klempnauer, Florian W. R. Vondran, Ulf Kulik

**Affiliations:** 1 Department of General, Visceral and Transplantation Surgery, Hannover Medical School, Carl-Neuberg Str. 1, 30625 Hannover, Germany; 2 Institute for Transfusion Medicine and Transplant Engineering, Hannover Medical School, Hannover, Germany

**Keywords:** Colorectal cancer, Liver metastases, Liver resection, Transfusion, Kidney function

## Abstract

**Background and aims:**

Recent studies focusing on thoracic surgery suggest postoperative kidney injury depending on the amount of perioperative blood transfusions. Data investigating similar effects after resection of colorectal liver metastases (CRLM) are not available. Aim of this study was therefore to evaluate the influence of perioperative blood transfusions on postoperative renal function and survival after resection of CRLM.

**Methods:**

Seven hundred twenty-seven cases of liver resection for CRLM were retrospectively analyzed. Renal function was measured via estimated glomerular filtration rate (eGFR) and a postoperative decline of ≥ 10% was considered substantial. Potential influences on postoperative kidney function were assessed using univariable and multivariable logistic regression analyses. Cox-regression analyses were performed to estimate the impact on overall survival (OS).

**Results:**

Preoperative impaired kidney function (*p* = 0.001, OR 2.477) and transfusion of > 2 units of packed red blood cells (PRBC) (*p* = 0.046; OR 1.638) were independently associated with an increased risk for ≥ 10% loss of renal function. Neither a pre-existing renal impairment, nor the additional loss of renal function were associated with reduced survival. Chemotherapies in the context of primary colorectal cancer treatment (*p* = 0.002), age > 70 years at liver resection (*p* = 0.005), number (*p* = 0.001), and size of metastases > 50 mm (*p* = 0.018), duration of resection > 120 min (*p* = 0.006) and transfusions of > 2 units of PRBC (*p* = 0.039) showed a negative independent influence on OS.

**Conclusion:**

The results demonstrate a negative impact of perioperative blood transfusions on the postoperative renal function and OS. Hence, efforts to reduce blood transfusions should be intensified.

## Introduction

Colorectal cancer (CRC) was the third most common cancer worldwide in 2020, with 1,931,590 new diagnosed cases [1]. Up to 30% of patients present themselves with either synchronous colorectal liver metastases (CRLM) at primary diagnosis or develop metachronous hepatic lesions in the following years [2]. The treatment of CRLM requires collaboration of oncologists, radiologists, and surgeons and comprises several different modalities. Depending on size, number, and location of hepatic lesions, liver function, pre-existing liver pathologies and previous therapies different treatment options are available. Surgical resection of CRLM, microwave or radiofrequency ablation, systemic chemotherapy and/or radiation can be applied. However, if technically and functionally possible, liver resection for CRLM with curative intent remains the treatment of choice and offers 5-year survival rates of up to 30% [3]. Only in case of reduced liver function and demands of a larger postoperative liver remnant interventional therapies represent viable primary treatment alternatives. To better estimate the individual chances of long-term survival, several risk factors that influence the oncological outcome have been identified over the last decades. Usually, a high number and large diameter of metastatic lesions, positive resection margins, extrahepatic disease, preoperative anemia, and higher age are considered to be associated with increased risk for impaired overall survival (OS) [4, 5].

Nevertheless, the postoperative mortality after liver resection for CRLM has improved over the last decades and averages between 1.5% and 5.5% [4, 6]. On the contrary, postoperative morbidity in general remains relatively high with up to 30% [7]. Frequent complications are biliary leakage, bilioma, and transient liver function impairment with subsequent need for coagulation substitution or blood transfusions [8]. In fact, some centers report that up to one third of resected patients require blood transfusions with different extent [9]. Over the past years many studies have reported inferior OS in case of intensive intra- or postoperative transfusions of packed red blood cells (PRBC) [10–12]. Negative effects of blood transfusions have been described for various tumor entities such as esophageal cancer, hepatocellular carcinoma, non-small-cell lung cancer, and urothelial carcinoma [13–16]. The mechanisms that underline these effects are not completely understood but many studies suggest a decreased immune function, called transfusion-related immunomodulation (TRIM) [9, 17, 18].

In addition to potential inferior OS, studies from thoracic surgery suggest that transfusion of PRBC might also impair the postoperative renal function [19]. Until now, data evaluating a potential association of blood transfusion requirements on the kidney function after liver resection for CRLM is surprisingly limited. However, a stable and adequate kidney function measured via the estimated glomerular filtration rate (eGFR) is an essential condition, not only with regard to the general quality of life, but also of major importance for future treatment options of a malignant disease. Many chemotherapeutical agents require a sufficient renal function due to renal drug elimination or general nephrotoxicity of the agents. For instance, antibodies targeting the vascular endothelial growth factor (anti-VEGF) are potentially nephrotoxic and need dose reduction in case of pre-existing renal diseases [20]. Furthermore, Nolin et al. demonstrated that kidney function impairments compromise not only the primary renal drug excretion, but also hepatic drug elimination pathways, which emphasizes the necessity of dose adjustment for patients with reduced renal function [21]. Hence, kidney insufficiency might become an important issue after liver resection for CRLM, especially in case of recurrent hepatic lesions with limited surgical treatment options and need for systemic chemotherapy. Moreover, other studies showed that patients, who suffered from an acute kidney injury after liver resection were at higher risk to develop a renal dysfunction within the following years [22]. Another study from the field of cardiac and thoracic surgery revealed that a postoperative temporary and persistent decrease of the renal function is more often associated with the development of chronic kidney disease in comparison with patients who did not have a postoperative decrease of renal function [23, 24].

Therefore, the aim of this study was to evaluate the frequency of a relevant kidney function impairment following liver resection for CRLM and a potential association with the amount of perioperativley transfused units of PRBC.

## Methods

### Study design and data collection

This is a single-center retrospective study from a German tertiary referral center for hepatobiliary surgery and liver transplantation. Included were all cases of primary liver resections for CRLM between 1 January 2000 and 31 December 2018. Liver resections of recurrent metastases were excluded. The follow-up ended on 1 January 2020. During the whole study period, liver resections for CRLM were performed with high personal continuity by few highly experienced surgeons.

The renal function prior hepatic surgery and at hospital discharge was estimated via the eGFR. The eGFR was calculated using the CKD-Epi equation on the basis of serum creatinine levels. Afterwards, the loss of renal function in comparison to preoperative values was computed. Mean loss of renal function was 90.1% of the preoperative eGRF; therefore, a substantial loss of eGFR was defined as 10%. Subsequently, the collective was divided in cases with and without substantial decrease of eGFR. Additionally, variables with possible impact on the eGFR or the OS, e.g., data on primary tumor and liver metastases, surgical procedure, and perioperative course were obtained from clinical documentation systems and office charts. Of note, patients that died during the initial hospital stay (in-hospital mortality) were also excluded from the analyses.

### Study end-points

The primary study end-point was defined as postoperative decrease of eGFR ≥ 10% at hospital discharge. The secondary end-point was OS in years.

### Statistical methods

Possible differences of clinical variables in the groups with or without impaired postoperative renal function were assessed using *t* tests, Mann-Whitney *U* tests or Kruskall-Wallis tests, as appropriate.

Variables were then included in a univariable binary logistic regression analysis based on clinically purposeful selection as recommended and published by Hosmer et al. [25]. Subsequently, all variables that showed a potential influence on the postoperative renal function (*p* < 0.300) were then submitted to multivariable regression analysis in a stepwise forward likelihood elimination until only significant variables remained in the regression model.

In a next step, uni- and multivariable Cox-regression analyses were performed to determine impact of variables on OS. Again, variables were included based on the principles of clinically purposeful variable selection published by Hosmer et. al. and variables with a *p* value of < 0.300 in the univariable analysis were considered in the multivariable Cox-regression analyses.

For all statistical tests a *p* value < 0.050 was defined as statistically significant. The statistical analyses were performed using the software SPSS version 24.0 (IBM, Somers, NY, USA). The grading of postoperative complications was performed according to Dindo-Clavien [26].

## Results

### Outcome parameters

A total of 775 patients with primary liver resection of CRLM were initially included into this study. The mean observed OS was 3.3 years and the median follow-up was 10.1 years. The overall postoperative morbidity was 30.7% and 12.8% of cases developed complications graded ≥ 3 according to Dindo-Clavien. Only 19 patients died after the surgery, representing a postoperative mortality of 2.5%. The majority of those cases developed a progressive post-hepatectomy liver failure (*N* = 8; 42.1%) or a septic multiple organ failure due to pneumonia or biliary tract complications (*N* = 5; 26.3%). Three (15.8%) patients died after cardiac complications and 2 (10.5%) cases developed intracranial bleeding. One (5.3%) patient suffered from massive intestinal bleeding and subsequent multiple organ failure.

In 29 cases (3.8%) data on the renal function was not available, therefore 727 cases were included in the final analysis. Of these, 81 cases (10.7%) developed a reduced kidney function with a ≥ 10% impairment of the eGFR. Table 1 depicts an overview of the selected outcome parameters.

**Table 1:** Summary of relevant outcome parameters after liver resection for CRLM

Outcome parameter ***N*** = 775 primary included cases	***N*** (%)/median (range)	Missing values
**Postoperative morbidity**	238 (30.7%)	0 (0%)
**Postoperative morbidity graded Dindo-Clavien ≥ 3**	99 (12.8%)	0 (0%)
**Postoperative mortality**	19 (2.5%)	0 (0%)
**Hospital stay (day)**	12.0 (5–109)	4 (0.5%)
**ICU-stay (day)**	1.0 (0–107)	13 (17%)
**≥ 10% decrease of preoperative renal function (eGFR < 90%)**	81 (10.7%)	29 (3.8%)
**Overall survival (OS) (years)**	3.3 (0.01–20.6)	17 (2.2%)
**Follow-up of living cases (years)**	10.4 (1.9–20.6)	17 (10.4%)

### Descriptive statistics

A descriptive summary of both cohorts (i.e., with or without postoperative impaired renal function) is depicted in Table 2. Of note, in the group of patients that displayed a loss of ≥ 10% eGFR at hospital discharge, mean eGFR values were already significantly lower at the time of admission and prior liver surgery. In fact, the frequency of patients with eGFR values below the lower reference limit was significantly higher in that respective cohort. Furthermore, patient age, the duration of the surgery, the percentage of patients with synchronous metastases and the transfusion requirements of packed red blood cells (PRBC) appeared to be higher in patients that developed kidney function impairments after hepatic resection. Also, the overall frequency of complications was elevated in the group with subsequent renal impairment, whereas the incidence of severe complications (Dindo-Clavien ≥ 3) was similar. All other variables were similarly distributed between both groups.

**Table 2:** The frequencies of included variables in both the cohort with impaired and not affected renal function

Variables	eGFR ≤ 90% of preoperative level ***N*** = 81	Missing values	eGFR > 90% of preoperative level ***n*** = 646	Missing values	***P*** value
**Pre-operative variables**					
Female gender	31 (38.3%)	0 (0%)	254 (39.3%)	0 (0%)	0.856^a^
Male gender	50 (61.7%)	0 (0%)	392 (60.7%)	0 (0%)	
UICC I	6 (9.0%)	14 (17.3%)	51 (7.9%)	92 (14.2%)	0.901^b^
UICC IIa	14 (17.3%)		90 (13.9%)		
UICC IIb	0 (0%)		8 (1.2%)		
UICC IIc	0 (0%)		0 (0%)		
UICC IIIa	2 (2.5%)		17 (2.6%)		
UICC IIIb	7 (8.6%)		78 (12.1%)		
UICC IIIc	3 (3.7%)		24 (3.7%)		
UICC IV	35 (82.7%)		554 (85.8%)		
Chemotherapy of primary tumor	53 (65.4%)	2 (2.5%)	427 (66.1%)	9 (1.4%)	0.992^a^
Time to metastases (years)	1.27 (0–17)	4 (4.9%)	1.34 (0–14)	12 (1.9%)	0.785^c^
Synchronous metastases (%)	35 (43.2%)	2 (2.5%)	250 (38.7%)	7 (1.1%)	0.375^a^
Number of CRLM	2.5 (1–10)	1 (1.2%)	2.4 (1–11)	11 (1.7%)	0.590^c^
Age at liver resection (years)	65.3 (26–87)	0%	61.7 (24–90)	0%	**0.005**^**c**^
Platelets Tsd/μl (NR 160–370)	250 (96–794)	1 (1.2%)	244 (15–977)	4 (0.6%)	0.548^c^
Hemoglobin g/dl (NR 11.8–15.8)	13.4 (9.1–16.8)	0%	13.4 (8.2–18.3)	4 (0.6%)	0.857^c^
Quick % (NR 70–130)	99.3 (63–137)	1 (1.2%)	99.1 (10–145)	8 (1.2%)	0.904^c^
Preoperative eGFR (NR 90–130 ml/min)	84.9 (40.3–150.8)	0 (0%)	89.4 (4.9–130.2)	0 (0%)	**0.022**^**c**^
Preoperative eGFR < 90 ml/min	56 (69.1%)		307 (47.5%)		**< 0.001**^**a**^
**Operative details and characteristics of metastases**					
Minor liver resection	45 (55.6%)	0 (0%)	372 (57.6%)	0 (0%)	0.728^a^
Major liver resection	36 (44.4%)		274 (42.4%)		
Operative duration in min	174 (40–385)	0 (0%)	163 (40–606)	4 (0.6%)	0.114^c^
Use of Pringle’s maneuver (in *N* cases)	25 (30.9%)	3 (3.7%)	201 (31.1%)	27 (4.2%)	0.963^a^
Pringle’s procedure in min if used	24.4 (7–72)		23.3 (4–63)		0.465^c^
Intraoperative transfusion of PRBC	1.7 (0–13)	0 (0%)	1.2 (0–42)	0 (0%)	0.125^c^
Postoperative transfusion of PRBC	1.2 (0–17)		0.8 (0–49)		0.266^c^
Overall transfusion of PRBC	2.9 (0–21)		2.0 (0–58)		0.089^c^
Size of largest metastasis in mm	44.7 (2–200)	3 (3.7%)	50.3 (4–220)	38 (5.9%)	0.175^c^
Weight of liver specimen in g	410 (2.2–2070)	3 (3.7%)	445 (2.8–4860)	34 (5.3%)	0.530^c^
Distance to resection margin in mm	4.4 (0–35)	5 (6.2%)	5.9 (0–70)	35 (5.4%)	0.139^c^
R-status R0	70 (86.4%)	1 (1.2%)	588 (91.0%)	8 (1.3%)	0.083^b^
R-status R1	9 (11.1%)		48 (7.4%)		
R-status R2	1 (1.2%)		2 (0.3%)		
eGFR at hospital dismission (Reference 90–130 ml/min)	64.5 (15.5–124-7)	0 (0%)	93.9 (6.5–138-7)	0 (0%)	**< 0.001**^**c**^
Dindo-Clavien classification of surgical complications grade 1	2 (2.5%)	0 (0%)	17 (2.6%)	0 (0%)	**0.005**^**b**^
Dindo-Clavien classification of surgical complications grade 2	17 (21.0%)		97 (15.0%)		
Dindo-Clavien classification of surgical complications grade 3	11 (13.6%)		67 (10.4%)		
Dindo-Clavien classification of surgical complications grade 4	6 (7.4%)		14 (2.2%)		
Complications graded Dindo-Clavien > 3	17 (21%)		81 (12.6%)		0.065^a^

Data presented in *N* (%) or mean (ranges)

*NR* Normal range

^a^Chi-square test

^b^Kruskal-Wallis test

^c^*t* test

### Logistic regression analysis—impact on postoperative renal function

Univariable logistic regression analyses indicated a possible influence of age (*p* = 0.005), pre-existing eGFR below lower reference limit (*p* < 0.001), transfusion of > 2 PRBC (*p* = 0.051), and complications graded Dindo-Clavien ≥ 3 (*p* = 0.068) on the development of a ≥ 10% loss of renal function after liver resection. The multivariable regression analysis demonstrated a pre-existing eGFR impairment (*p* = 0.001; OR 2.477) and transfusion of more than 2 PRBC (*p* = 0.046; OR 1.638) as independent risk factors for a decline in postoperative kidney function. All other variables including chemotherapies prior surgery, age > 70 years, the extent of the liver resection and surgical duration, the use of pedicle clamping (PC), and postoperative complications appeared to have no impact on the postoperative kidney function. Table 3 summarizes the results of the logistic regression analyses.

**Table 3:** Results of univariable biniary and multivariable logistic regression analyses of variables with possible influence on 10% impairment of the postoperative eGFR

Variables	Univariable binary logistic regression		Multivariable logistic regression	
	***p*** value	OR (95%-CI)	***p*** value	OR (95%-CI)
**Preoperative variables**				
Chemotherapy on primary tumor (Ref. yes)	0.992	1.003 (0.610–1.649)	Not included	
Development of CRLM (Ref. metachronous)	0.375	0.808 (0.504–1.295)		
Age at liver resection (years)	0.005	1.034 (1.010–1.058)	0.085	Not applicable
Number of CRLM	0.590	1.029 (0.928–1.139)	Not included	
Size of CRLM	0.175	0.995 (0.987–1.002)	0.140	Not applicable
Preoperative hemoglobin	0.858	0.987 (0.852–1.142)	Not included	
Preoperative platelets	0.548	1.001 (0.998–1.003)		
Preoperative quick	0.901	1.001 (0.984–1.018)		
Preoperative eGFR < 90 ml/min (Ref. normal values)	< 0.001	2.466 (1.502–4.050)	**0.001**	2.477 (1.478–4.151)
**Intra- and postoperative variables**				
Extent of liver resection (Ref. minor)	0.728	1.086 (0.682–1.729)	Not included	
Operative duration > 120 min (Ref. no)	0.116	1.003 (0.999–1.006)	0.548	Not applicable
Use of Pringle maneuver (Ref. not used)	0.963	1.012 (0.614–1.668)	Not included	
Weight of liver specimen in g	0.529	1.000 (0.999–1.000)		
Overall transfusion of > 2 units PRBC (Ref. no)	0.051	1.586 (0.998–2.522)	**0.046**	1.638 (1.010–2.657)
Postoperative complications graded Dindo-Clavien > 3 (Ref. no)	0.068	1.742 (0.961–3.157)	0.451	Not applicable
ICU-stay (days)	0.298	1.017 (0.986–1.048)	0.790	

*OR* Odds ratio, *CI* Confidence intervall

### Cox-regression analysis—survival after liver resection

The univariable Cox-regression analyses suggested an association with impaired OS in case of administration of chemotherapies during treatment of the primary colorectal cancer (*p* < 0.001), age > 70 years (*p* = 0.009), number of CRLM (*p* < 0.001), size of the CRLM > 50 mm (*p* < 0.001) and pre-existing eGFR below normal range (*p* = 0.036). Furthermore, performance of major liver resections (*p* = 0.038), a duration of the surgery > 120 min (*p* < 0.001), overall transfusion requirements of > 2 PRBC (*p* < 0.001), longer stay on the ICU (*p* < 0.001), and overall hospital stay >14 days (*p* < 0.001) appeared to limit the OS after liver resection for CRLM. On the contrary, the width of the resection margin showed a positive influence on the outcome after surgical treatment of CRLM (*p* = 0.005). The use of PC, impaired renal function of ≥ 10% compared to preoperative values, complications graded Dindo-Clavien ≥ 3 and, interestingly, the resection status itself showed no impact on the OS.

**Fig. 1:**
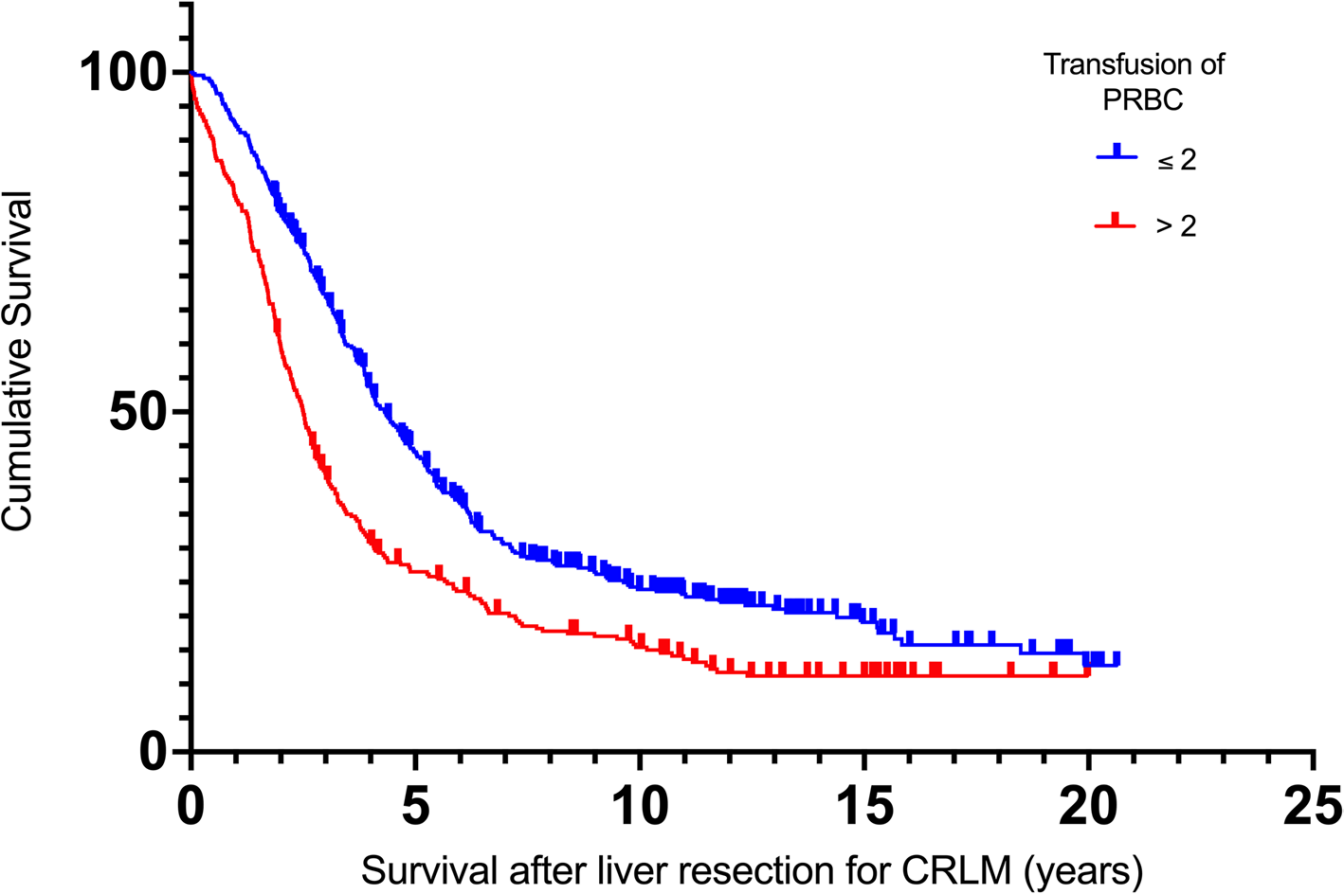
Kaplan-Meier curve regarding survival after liver resection for CRLM depending on transfusion of > 2 PRBC. *P* < 0.001

In the multivariable Cox-regression analysis, a statistically independent effect on the OS was evident for seven variables: chemotherapies administered for treatment of the primary colorectal disease, age > 70 years at liver resection, number of hepatic lesions and size > 50 mm, duration of resections > 120 min and overall transfusions of > 2 units of PRBC showed a negative influence on OS. Figure 1 depicts the overall survival depending on the transfusion of > 2 PRBC. Also, the outcome was statistically superior in case of a wider resection margin proven by histopathology. Neither pre-existing renal function alterations nor a postoperative decrease of eGFR influenced the OS. Table 4 shows a summary of the results of both the univariable and multivariable Cox-regression analyses with all *p* values, hazard ratios and the appendant confidence intervals.

**Table 4:** Results of the Cox regression analyses and multivariable Cox regression analyses on variables with impact on overall survival. In-house mortality cases were excluded

Variables	Univariable Cox regression		Multivariable Cox regression	
	***p*** value	HR (95%-CI)	***p*** value	HR (95%-CI)
**Preoperative variables**				
Chemotherapy on primary tumor (Ref. yes)	< 0.001	0.722 (0.604–0.863)	**0.002**	0.732 (0.603–0.888)
Development of CRLM (Ref. metachronous)	0.101	1.151 (0.973–1.361)	0.835	Not applicable
Age at liver resection > 70 years	0.009	1.290 (1.066–1.562)	**0.005**	1.357 (1.095–1.683)
Number of CRLM	< 0.001	1.115 (1.076–1.156)	**0.001**	1.071 (1.028–1.115)
Size of CRLM > 50 mm	< 0.001	1.416 (1.200–1.671)	**0.018**	1.256 (1.040–1.517)
Preoperative hemoglobin	0.017	0.939 (0.891–0.989)	0.683	Not applicable
Preoperative platelets	0.148	1.001 (1.000–1.002)	0.339	
Preoperative quick	0.127	0.996 (0.990–1.001)	0.826	
Preoperative eGFR < 90 ml/min (Ref. normal values)	0.036	1.196 (1.012–1.413)	0.113	
**Intra- and postoperative variables**				
Extent of liver resection (Ref. minor)	0.038	1.190 (1.009–1.404)	0.830	
Operative duration > 120 min (Ref. no)	< 0.001	1.460 (1.192–1.788)	**0.006**	1.385 (1.096–1.749)
Use of Pringle’s maneuver (Ref. not used)	0.380	1.038 (0.907–1.293)	0.643	Not applicable
Weight of liver specimen > 450 g	0.062	1.170 (0.992–1.380)	0.295	
Distance to resection margin in mm	0.005	0.984 (0.973–0.995)	**0.041**	0.988 (0.977–1.000)
R1-status (Ref. R0)	0.466	1.129 (0.815–1.564)	0.425	Not applicable
R2-status (Ref. R0)	0.261	1.658 (0.687–4.002)	0.440	
Overall transfusion of > 2 units PRBC (Ref. no)	< 0.001	1.468 (1.243–1.734)	**0.039**	1.223 (1.010–1.482)
Postoperative complications graded Dindo-Clavien > 3 (Ref. no)	0.063	1.259 (0.988–1.605)	0.285	Not applicable
eGFR at dismission < 90% of preoperative eGFR (Ref. < 90%)	0.494	1.096 (0.843–1.423)	0.915	
Stay on intensive care unit (days)	< 0.001	1.029 (1.016–1.042)	0.122	
Overall hospital stay > 14 days (Ref.no)	< 0.001	1.382 (1.162–1.643)	0.190	

*HR* Hazard ratio

## Discussion

This study was initiated to evaluate a potential influence of perioperative blood transfusions on the postoperative renal function and OS after liver resection for CRLM. The presented data clearly emphasizes that blood transfusions independently affect the OS (*p* = 0.039; HR 1.223). This work is also the first to evidence that perioperative transfusion of more than two PRBC is independently associated with increased risk of renal function impairments after liver resection for CRLM (*p* = 0.0; OR).

The negative effect of blood transfusions on the perioperative morbidity, mortality and the overall oncological outcome was widely discussed over the last decades, but data derived from large studies were controversial. Nevertheless, although some studies reported no association of transfusions and OS, numerous publications support the findings of the present study and provided strong evidence that transfusion of PRBC is associated with inferior disease-free and overall survival after liver resection for CRLM [9, 27–29]. Moreover, a recent study could also demonstrate higher risks for postoperative morbidity depending on intraoperative transfusion requirements [30]. These findings are supported by a population-based study from the USA that reported increased risk for in-hospital mortality, morbidity and longer hospital stay [10]. A systematic review by Acheson et al. recommended restrictive use of PRBC in surgeries of primary colorectal cancer (CRC) due to negative impacts on clinical parameters such as overall and cancer-related mortality [31]. It was also described that perioperative blood replacements in patients with CRC are associated with higher rate of postoperative infections and morbidity [32].

Nevertheless, all those studies disregarded effects on the renal outcome after resection of CRLM evidenced in the presented study. The result appears to be of substantial importance, especially since approximately 50% (*N* = 363) of all patients included in the study already showed pre-existing renal function impairments prior the liver resection. In those cases, the risk for an additional loss of renal function capacity was statistically independently elevated. The underlying pathophysiological mechanisms were not subject of this study. However, it is discussed that renal hypoperfusion related to liver function impairments after resection result in clinically apparent renal injury of pre-existing renal dysfunction [33].

Interestingly, neither a pre-existing kidney injury, nor an additional loss of renal capacity appear to directly influence the OS. Presumably, the underlying effects of blood transfusions develop stronger impact on survival, possibly via immunological mechanisms that are not yet understood completely. A possible explanation might be toxic effects of circulating free heme that was shown to be elevated in critical ill patients after transfusion of PRBC and might contribute to oxidative stress, membrane, and cell injury, possibly resulting in renal impairment [34–36].

Nonetheless, a potential kidney injury related to blood transfusions could still inherit implications for the further treatment of patients suffering from CRLM, especially in case of recurrent hepatic or even extrahepatic disease with need for systemic chemotherapy [37, 38]. Accordingly, it was shown that reduced renal function increases the risk to suffer from oxaliplatin-related nausea with the necessity of dose adjustment or even discontinuation of treatment [33].

Moreover, age > 70 years was again proven to be associated with limited survival. It must be assumed that patients of higher age more often present themselves with underlying renal diseases, other co-morbidities and a limited general condition [39]. Hence, statistical effects of renal function impairment on OS could be masked by confounding variables such as higher age and necessity of blood transfusions. In conclusion, it appears obvious that strategies to reduce transfusion requirements, particularly in case of renal co-morbidities need to be improved. One option to limit the use of PRBC is to enhance hemoglobin levels perioperatively via the substitution of erythropoietin, although a study from 2009 which also included patients undergoing surgical resection of primary colorectal cancer could not derive a recommendation for such a procedure [40]. More promising could be the consequent use of PC to reduce blood loss during the parenchyma dissection. Often only applied based on the surgeon’s discretion in case of bleeding, PC should be performed in a more preventive manner. Main reason for a limited use was usually to avoid warm ischemia in the hepatic tissue and risk of impaired liver function after the liver surgery, mostly in case of major hepatic resections and pre-existing liver damage [41, 42]. Unfortunately, the majority of studies exploring the effects of PC on the postoperative outcome, including this study, were designed retrospectively and the decision-making when to use PC is often vague and highly individual. Nevertheless, based on recent studies and the presented data, the use of PC appears not to influence the postoperative morbidity, mortality and overall survival [42]. One published study even reported improved oncological outcome when PC was performed [43]. Therefore, if the quality of the hepatic tissue tolerates performance of PC it should be applied more frequently, especially if the renal capacity is already impaired prior liver surgery. Additionally, prospective studies for a more detailed analysis of the effects of PC are indicated.

Main limitation of this study is the retrospective nature, the single-center design and given that, the lack of data on co-morbidities. Furthermore, information on cancer recurrence and the exact cause of death could not be obtained and only impacts on the overall survival were to analyze.

In summary, this is the first study to present evidence that the transfusion of more than two units of PRBC is an independent risk factor for the loss of renal function after liver resection for CRLM—a finding that implies intensified measures to avoid blood loss and transfusion during hepatic metastasectomy, especially in patients suffering from pre-existing renal insufficiency. Most promising instrument might be the more frequent and standardized use of PC during parenchyma dissection and prospective studies on this matter are highly desirable.

## Data Availability

The datasets used and analyzed during the current study are available from the corresponding author on reasonable request.

## Abbreviations

CRC: Colorectal cancer
CRLM: Colorectal liver metastasis
eGFR: Estimated glomerular filtration rate
OS: Overall survival
PC: Pedicle clamping
PRBC: Packed red blood cells
TRIM: Tranfusion-related immunomodulation
OR: Odds ratio
HR: Hazard ratio
NR: Normal range
CI: Confidence interval

## References

[1] International Agency for Research on Cancer, (IARC). Colorectal cancer. Available at: https://gco.iarc.fr/today/fact-sheets-cancers. Accessed 4 May 2021.

[2] Engstrand J, Nilsson H, Strömberg C, Jonas E, Freedman J (2018). Colorectal cancer liver metastases – a population-based study on incidence, management and survival. BMC Cancer.

[3] Kato T, Yasui K, Hirai T, Kanemitsu Y, Mori T, Sugihara K (2003). Therapeutic results for hepatic metastasis of colorectal cancer with special reference to effectiveness of hepatectomy: analysis of prognostic factors for 763 cases recorded at 18 institutions. Dis Colon Rectum.

[4] Rees M, Tekkis PP, Welsh FK, O'Rourke T, John TG (2008). Evaluation of long-term survival after hepatic resection for metastatic colorectal cancer: a multifactorial model of 929 patients. Ann Surg.

[5] Gwiasda J, Schrem H, Kaltenborn A, Mahlmann J, Mix H, Lehner F (2017). Introduction of the resection severity index as independent risk factor limiting survival after resection of colorectal liver metastases. Surg Oncol.

[6] Filmann N, Walter D, Schadde E, Bruns C, Keck T, Lang H (2019). Mortality after liver surgery in Germany. Br J Surg.

[7] Laurent C, Sa Cunha A, Couderc P, Rullier E, Saric J (2003). Influence of postoperative morbidity on long-term survival following liver resection for colorectal metastases. Br J Surg.

[8] Jin S, Fu Q, Wuyun G, Wuyun T (2013). Management of post-hepatectomy complications. World J Gastroenterol.

[9] Schiergens TS, Rentsch M, Kasparek MS, Frenes K, Jauch KW, Thasler WE (2015). Impact of perioperative allogeneic red blood cell transfusion on recurrence and overall survival after resection of colorectal liver metastases. Dis Colon Rectum.

[10] Long B, Xiao Z, Shang L, Pan B, Chai J (2019). Impact of perioperative transfusion in patients undergoing resection of colorectal cancer liver metastases: a population-based study. World J Clin Cases.

[11] Nanji S, Mir ZM, Karim S, Brennan KE, Patel SV, Merchant SJ (2021). Perioperative blood transfusion and resection of colorectal cancer liver metastases: outcomes in routine clinical practice. HPB (Oxford).

[12] Lyu X, Qiao W, Li D, Leng Y (2017). Impact of perioperative blood transfusion on clinical outcomes in patients with colorectal liver metastasis after hepatectomy: a meta-analysis. Oncotarget.

[13] Boshier PR, Ziff C, Adam ME, Fehervari M, Markar SR, Hanna GB. Effect of perioperative blood transfusion on the long-term survival of patients undergoing esophagectomy for esophageal cancer: a systematic review and meta-analysis. Dis Esophagus. 2018;31(4). 10.1093/dote/dox134. 10.1093/dote/dox134 29267869

[14] Shiba H, Ishida Y, Wakiyama S, Iida T, Matsumoto M, Sakamoto T (2009). Negative impact of blood transfusion on recurrence and prognosis of hepatocellular carcinoma after hepatic resection. J Gastrointest Surg.

[15] Sakin A, Sahin S, Yasar N, Demir C, Arici S, Geredeli C (2019). Prognostic impact of blood transfusion in patients with metastatic non-small cell lung cancer receiving chemotherapy. Lung Cancer.

[16] Buchner A, Grimm T, Schneevoigt BS, Wittmann G, Kretschmer A, Jokisch F (2017). Dramatic impact of blood transfusion on cancer-specific survival after radical cystectomy irrespective of tumor stage. Scand J Urol.

[17] McSorley ST, Tham A, Dolan RD, Steele CW, Ramsingh J, Roxburgh C (2020). Perioperative blood transfusion is associated with postoperative systemic inflammatory response and poorer outcomes following surgery for colorectal cancer. Ann Surg Oncol.

[18] Aguilar-Nascimento JE, Zampieri-Filho JP, Bordin JO (2021). Implications of perioperative allogeneic red blood cell transfusion on the immune-inflammatory response. Hematol Transfus Cell Ther.

[19] Shimmer C, Hamouda K, Ozkur M, Sommer SP, Hain J, Aleksic I (2013). Influence of storage time and amount of red blood cell transfusion on postoperative renal function: an observational cohort study. Heart Lung Vessels.

[20] Launay-Vacher V, Aapro M, De Castro G, Cohen E, Deray G, Dooley M (2015). Renal effects of molecular targeted therapies in oncology: a review by the Cancer and the Kidney International Network (C-KIN). Ann Oncol.

[21] Nolin TD, Naud J, Leblond FA, Pichette V (2008). Emerging evidence of the impact of kidney disease on drug metabolism and transport. Clin Pharmacol Ther.

[22] Ishikawa S, Tanaka M, Maruyama F, Fukagawa A, Shiota N, Matsumura S (2017). Effects of acute kidney injury after liver resection on long-term outcomes. Korean J Anesthesiol.

[23] Kuijk J, Flu W, Chonchol M, Hoeks S, Winkel T, Verhagen H (2010). Temporary perioperative decline of renal function is an independent predictor for chronic kidney disease. Clin J Am Soc Nephrol.

[24] Rouer M, Monnot A, Bubenheim M, Fuda M, Godier S, Lebras M (2020). Early postoperative renal dysfunction predicts long-term renal function degradation after type IV thoracoabdominal aortic aneurysm surgical repair. Ann Vasc Surg.

[25] Hosmer DW, Lemeshow S (2013). Sturdivant RX applied Logistic regression.

[26] Dindo D, Demartines N, Clavien P (2004). Classification of surgical complications: a new proposal with evaluation in a cohort of 6336 patients and results of a survey. Ann Surg.

[27] Hallet J, Tsang M, Cheng ES, Habashi R, Kulyk I, Hanna SS (2015). The impact of perioperative red blood cell transfusions on long-term outcomes after hepatectomy for colorectal liver metastases. Ann Surg Oncol.

[28] Pathak S, Al-Duwaisan A, Khoyratty F, Lodge JPA, Toogood GJ, Salib E, et al. Impact of blood transfusion on outcomes following resection for colorectal liver metastases in the modern era. ANZ J Surg. 2018. 10.1111/ans.14257. 10.1111/ans.14257 29961953

[29] Kang R, Seath BE, Huang V, Barth RJ (2019). Impact of autologous blood transfusion on survival and recurrence among patients undergoing partial hepatectomy for colorectal cancer liver metastases. J Am Coll Surg.

[30] Sneidere M, Schrem HH, Mahlmann JC, Beetz O, Cammann S, Oldhafer F, et al. Proposal of a multivariable prediction model for graded morbidity after liver resection for colorectal metastases. Zentralbl Chir. 2020. 10.1055/a-1243-0746. 10.1055/a-1243-0746 33091938

[31] Acheson AG, Brookes MJ, Spahn DR (2012). Effects of allogeneic red blood cell transfusions on clinical outcomes in patients undergoing colorectal cancer surgery: a systematic review and meta-analysis. Ann Surg.

[32] Mynster T, Christensen IJ, Moesgaard F, Nielsen HJ (2000). Effects of the combination of blood transfusion and postoperative infectious complications on prognosis after surgery for colorectal cancer. Danish RANX05 Colorectal Cancer Study Group. Br J Surg.

[33] Betrosian AP, Agarwal B, Douzinas EE (2007). Acute renal dysfunction in liver diseases. World J Gastroenterol.

[34] Pietropaoli AP, Henrichs KF, Cholette JM, Spinelli SL, Phipps RP, Refaai MA (2019). Total plasma heme concentration increases after red blood cell transfusion and predicts mortality in critically ill medical patients. Transfusion.

[35] Tracz MJ, Alam J, Nath KA (2007). Physiology and pathophysiology of heme: implications for kidney disease. J Am Soc Nephrol.

[36] Chiabrando D, Vinchi F, Fiorito V, Mercurio S, Tolosano E (2014). Heme in pathophysiology: a matter of scavenging, metabolism and trafficking across cell membranes. Front Pharmacol.

[37] Watanabe D, Fujii H, Yamada Y, Iihara H, Ishihara T, Matsuhashi N (2020). Relationship between renal function and the incidence of adverse events in patients with colorectal cancer receiving oxaliplatin. Anticancer Res.

[38] Launay-Vacher V, Oudard S, Janus N, Gligorov J, Pourrat X, Rixe O (2007). Prevalence of renal insufficiency in cancer patients and implications for anticancer drug management: the renal insufficiency and anticancer medications (IRMA) study. Cancer.

[39] Denic A, Glassock RJ, Rule AD (2016). Structural and functional changes with the aging kidney. Adv Chronic Kidney Dis.

[40] Devon KM, McLeod RS. Pre and peri-operative erythropoietin for reducing allogeneic blood transfusions in colorectal cancer surgery. Cochrane Database Syst Rev. 2009;(1):CD007148. 10.1002/14651858.CD007148.pub2. 10.1002/14651858.CD007148.pub2 19160325

[41] Matsuda A, Miyashita M, Matsumoto S, Matsutani T, Sakurazawa N, Akagi I (2013). Hepatic pedicle clamping does not worsen survival after hepatic resection for colorectal liver metastasis: results from a systematic review and meta-analysis. Ann Surg Oncol.

[42] Teoh NC (2011). Hepatic ischemia reperfusion injury: contemporary perspectives on pathogenic mechanisms and basis for hepatoprotection—the good, bad and deadly. J Gastroenterol Hepatol.

[43] Schiergens TS, Drefs M, Dörsch M, Kühn F, Albertsmeier M, Niess H (2020). Prognostic impact of pedicle clamping during liver resection for colorectal metastases. Cancers.

